# *Mycobacterium tuberculosis* protein MoxR1 enhances virulence by inhibiting host cell death pathways and disrupting cellular bioenergetics

**DOI:** 10.1080/21505594.2023.2180230

**Published:** 2023-02-26

**Authors:** Neha Quadir, Mohd. Shariq, Javaid Ahmad Sheikh, Jasdeep Singh, Neha Sharma, Seyed Ehtesham Hasnain, Nasreen Zafar Ehtesham

**Affiliations:** a National Institute of Pathology, ICMR, Safdarjung Hospital Campus, New Delhi, India; b Institute of Molecular Medicine, Jamia Hamdard, Hamdard Nagar, New Delhi, India; c Department of Biotechnology, Jamia Hamdard, Hamdard Nagar, New Delhi, India; d Department of Biochemical Engineering and Biotechnology, Indian Institute of Technology, New Delhi, India; e Department of Life Science,School of Basic Science and Research, Sharda University, Greater Noida, India

**Keywords:** AAA+ family protein, chaperone, MAPK signalling, NFKB, proinflammatory cytokines, survival signalling

## Abstract

*Mycobacterium tuberculosis* (*M. tb*) utilizes the multifunctionality of its protein factors to deceive the host. The unabated global incidence and prevalence of tuberculosis (TB) and the emergence of multidrug-resistant strains warrant the discovery of novel drug targets that can be exploited to manage TB. This study reports the role of *M. tb* AAA+ family protein MoxR1 in regulating host-pathogen interaction and immune system functions. We report that MoxR1 binds to TLR4 in macrophage cells and further reveal how this signal the release of proinflammatory cytokines. We show that MoxR1 activates the PI3K-AKT-MTOR signalling cascade by inhibiting the autophagy-regulating kinase ULK1 by potentiating its phosphorylation at serine 757, leading to its suppression. Using autophagy-activating and repressing agents such as rapamycin and bafilomycin A1 suggested that MoxR1 inhibits autophagy flux by inhibiting autophagy initiation. MoxR1 also inhibits apoptosis by suppressing the expression of MAPK JNK1/2 and cFOS, which play critical roles in apoptosis induction. Intriguingly, MoxR1 also induced robust disruption of cellular bioenergetics by metabolic reprogramming to rewire the citric acid cycle intermediates, as evidenced by the lower levels of citric acid and electron transport chain enzymes (ETC) to dampen host defence. These results point to a multifunctional role of *M. tb* MoxR1 in dampening host defences by inhibiting autophagy, apoptosis, and inducing metabolic reprogramming. These mechanistic insights can be utilized to devise strategies to combat TB and better understand survival tactics by intracellular pathogens.

## Introduction

One of the deadliest human diseases, tuberculosis, is caused by the highly adaptable intracellular bacteria *Mycobacterium tuberculosis* (*M. tb*) [1]. This extraordinary ability gained by *M. tb* is attributed to the evolution of protein moonlighting functions to compensate for reductive evolution [1–3]. Professional phagocytes such as macrophages, neutrophils, and dendritic cells serve as the primary defence arsenal against *M. tb* [4]. These phagocytes possess diverse pattern recognition receptors (PRRs), including toll-like receptors (TLRs) that are highly conserved, which enable them to recognize and start innate immunological reactions in response to different *M. tb* antigens [5–7]. A cascade of downstream signalling events is initiated through these activated TLRs that lead to the secretion of effector cytokines which regulate innate and adaptive immune responses [6–9]. The activation of the surface TLRs, such as TLR2 or TLR4, by the mycobacterial protein ligands, leads to the activation of the canonical nuclear factor kappa B (NFKB) and map kinase pathways (MAPK) [10–13].

*M. tb* has evolved an arsenal of virulence factors to modulate host cell death pathways. Autophagy and apoptosis are two crucial host innate defences implicated in controlling pathogenic micro-organisms, including intracellular pathogen *M. tb* [14–17]. Xenophagy is a form of selective autophagy in which diverse bacteria, viruses, and other pathogenic organisms are degraded by forming a vesicle called the autophagosome, which fuses with lysosomes to form autolysosome enriched with degradative enzymes such as hydrolases for killing the pathogens [18]. Apoptosis is considered an immunologically silent process crucial for clearing cells infected with *M. tb* [19,20]. *M. tb* exploits its virulence factors for spatiotemporal regulation of apoptosis for its efficient survival and dissemination [21].

MoxR1 (Rv1479) of *M. tb* is an ATP-dependent chaperone required for secreting RipA, a peptidoglycan hydrolase or endopeptidase using TAT (twin-arginine translocation) pathway [22]. We deciphered the function of MoxR1 in the pathophysiology of *M. tb* and its role in regulating host-pathogen interactions. The MoxR1 sequence analysis unveiled its selective interaction with TLR4 and the presence of the LIR (LC3-interacting region) motif, indicating its possible involvement in regulating host autophagy. Therefore, we postulated that MoxR1 might be involved in regulating various steps of the host autophagy, such as phagophore biogenesis, extension, maturation, and fusion to the lysosomes. The current study revealed MoxR1‘s involvement in controlling immune function and host-pathogen interaction. We show that MoxR1 is a ubiquitously expressed protein of *M. tb* that binds and activates TLR4 and NFKB signalling for heightened secretion of TNF, IL6, and IL12. Notably, MoxR1 inhibits autophagy, which depends on activating the pro-survival signalling pathway PI3K-AKT-MTOR, which culminates in repressing kinase ULK1, which performs a crucial function in autophagy initiation and phagophore biogenesis. Using pharmacological modulators of autophagy, we show that MoxR1 inhibits autophagy initiation mediated *via* TLR4. MoxR1 suppresses apoptosis by inhibiting the activity of transcription factors JNK1/2 and c-FOS. Interestingly, MoxR1 also induced metabolic reprogramming by inhibiting the production of the citric acid cycle and electron transport chain enzymes pyruvate dehydrogenase (PD), CoxIV, cytochrome C (CytC), and succinate dehydrogenase (SDHA). Pathogens have evolved an armoury for disrupting host cellular bioenergetics to dampen the host defences. The induction of metabolic repurposing is one of the attributes of pathogenic bacteria such as *Legionella pneumophila* and *M. tb* for efficient recycling of citric acid cycle intermediates for its utilization.

Our findings suggest that MoxR1 is a moonlighting protein of *M. tb*, which comprises multiple functions for efficient regulation of immune system function and hampering the host-directed defences. Our study highlights the essential functions of MoxR1 in *M. tb* pathophysiology, virulence, and pathogenesis that can be exploited to better understand the TB disease pathomechanism.

## Materials and methods

### Cloning of recombinant moxR1 in Escherichia coli (E. coli) and M. smegmatis

*M. tb*. genomic DNA was used to clone the *moxR1* (Rv1479) gene into *E. coli* and *M. tb* expression vectors such as pET-28a and pST-2K [22]. The list of bacterial strains and primers is listed in **Tables S1 and S2**. MoxR1 was expressed as a His-tag protein using the pET-28a vector. Minor changes were made to the previously used Ni-NTA (Nitriloacetic acid) chromatography protocol to purify the His-tagged MoxR1 protein [22]. pET-28a containing *moxR1* gene was expressed in ClearColi® BL21 (DE3) cells (Lucigen, BioSearch Technologies, USA) using 0.1 mM Isopropyl-β-D-thiogalactoside (IPTG) at 37^ο^C for 4 hrs until OD_600_nm reached 0.8–1. The purified protein was dialysed using the earlier described protocol [7]. Western blotting was used to check the purity of the recombinant protein (**Figures S1A and B**). The ClearColi® BL21 (DE3) cells are genetically modified to produce LPS that does not induce endotoxin response [23]. Protein concentration was estimated using the Bradford reagent (BioRad Laboratories, USA), and the absence of LPS in the purified protein sample was verified as described earlier USA) [24]. We did not detect any colour development after 2 hrs of incubation of the reagent with protein, which confirmed the absence of endotoxin contamination in the purified protein samples. An empty vector and *moxR1* cloned in pst-2K was transformed into competent *M. smeg* cells. The expression of the MoxR1 protein was verified by employing polyclonal αMoxR1 antibody at a dilution of 1:5000 (**Figure S1C**). *moxR1* containing positive clones of *M. smeg* were selected on an agar plate containing kanamycin (30 µg/ml). A single colony of recombinant *M. smeg* containing *moxR1* was used to grow and set up the experiments.

### Macrophage culture and growth condition

Mouse-originated wild-type (RAW264.7) and various ∆TLRs cell lines, including the MYD88 and MYD88/TRIF knockout cell lines, were used for cytokine estimation after treatment with MoxR1. ∆TLRs, MYD88, and MYD88/TRIF-knockout macrophages were obtained from the resources of the BEI (NIAID, NIH, USA). These macrophages were cultured in advanced DMEM containing antibiotic and foetal bovine serum. The cells were grown at 37°C with 5% CO2 in an incubator.

### Estimation of cytokines using ELISA

In 6 well plates, RAW264.7 (3 × 10^6^) macrophages were grown. MoxR1 protein was treated in cells at doses ranging from 1 µg/ml to 4 µg/ml. The negative control, Heat Inactivated protein (HI), was employed to completely rule out the possibility of LPS contamination. Proteinase K was used to completely digest the 4 µg/ml of MoxR1 protein at 60 °C for 4 hrs, followed by 2 hrs of heat inactivation at 100 °C. The positive control consisted of LPS (1 µg/ml). The supernatant was collected and kept at ultra-low temperature until needed. The cytokines were estimated using the kits procured from BD, Biosciences, USA.

### Analysis of apoptosis in macrophages

In a 24-well plate, RAW264.7 (0.5 × 10^6^) macrophage cells were plated. 4 µg/ml of MoxR1 was used to treat cells for 24 hrs at 37 °C. A positive control involving RAW264.7 cells was treated with 0.1 µM staurosporine (Sigma, USA) for 24 hrs. The ZVAD-FMK (20 µM) was used as a caspase inhibitor and served as the control for apoptosis inhibition. Untreated cells and cells treated with HI MoxR1 (4 µg/ml) served as the negative controls. The Annexin V/PI kit was used in detecting apoptosis. BD FACS Verse Flow Cytometer was used to measure the fluorescent intensity of the stained samples. Following sample reading acquisition, cells were examined using the FlowJo software.

### Western blot analysis of protein samples

RAW264.7 and TLR4-deficient (3 × 10^6^) macrophage cells were grown in culture plates. After 4 hrs of growth, cells were stimulated with proteins. Post-treatment completion, cells were rinsed with 1× PBS (RT) twice and treated with 2× SDS dye [25]. The protein was run in varying percentages of SDS-PAGE gel depending on the size of the proteins to be probed. The protein samples were transferred to the blotting membrane (PVDF) for western blot analysis. The membranes were then probed using the primary antibodies against the respective antigens, such as NFKB1, pP-65, cytochrome C (CytC), LC3BII, SQSTM1, beclin1, pMTOR, pULK1, pAKT, pPI3K, HSP60, pcFOS, tcFOS, pJNK1/2, tJNK1/2, tERK1/2, pERK1/2, tP38, pP38, pyruvate dehydrogenase (PD), succinate dehydrogenase (SDHA), CoxIV and Rab7, GAPDH and β-actin antibodies (Cell Signalling). The location of MoxR1 in various purified cellular materials of *M. tb* was investigated using the antibody against MoxR1 (αMoxR1) (BEI, Resources, NIAID, NIH, USA). The affinity-purified His-tagged MoxR1 protein was confirmed using a monoclonal mouse anti-His antibody (Sigma, USA). For signal generation, secondary antibodies were used as appropriate. Rapamycin and bafilomycin A1 treatment are used for 6 hrs along with MoxR1 treatment to explore its function in autophagy regulation. The protein samples were then prepared by lysis and processed [7,26]. The western blotting performed to detect the proteins onto the PVDF membrane. 5% BSA in TBST was used for blocking the PVDF membrane in the western blotting of phosphoproteins. The band intensities of proteins were quantified using ImageJ software. GAPDH, β-actin, tFos, tERK1/2, tP38, or tJNK1/2 was used to normalize protein bands as required.

### Production of anti-MoxR1 polyclonal antibody

The purified MoxR1 protein (His-tagged) of *M. tb* was used to induce the production of an αMoxR1 antibody in the rabbit. A 90-day-old female New Zealand rabbit was injected with 500 µg of emulsified MoxR1 protein and 500 µl of Freund’s incomplete adjuvant (500 µg MoxR1 protein in PBS pH-7.4 with 500 µl of adjuvant) was used in the first injection. Following the initial injection, 3-boosters were injected at 28 days. The rabbit was then bled through an ear vein. The serum was collected and frozen. Titre of αMoxR1 was determined using western blotting (**Figures S1C and D**).

### Ethics statement

The female New Zealand rabbits were approved by the ICMR National Institute of Pathology’s (ICMR-NIOP) Animal Ethics Committee to produce antibodies. As suggested by the committee members, the rabbit was kept in the animal facility of the ICMR-NIOP. The experiment was completed, and the rabbit was rehabilitated at AIIMS.

### Infection of macrophage cells with wild-type and recombinant *M. smegmatis*

In a 6-well culture plate, RAW264.7 macrophages (2 × 10^6^ cells/well) were grown. For adhesion, the cells were left at 37°C overnight. *M. smeg*-containing moxR1 gene and the pST-2K vector alone were cultured until the log phase (OD600nm was between 0.3 and 0.4). Log phase-grown *M. smeg* was collected on the day of infection, centrifuged, and washed. After washing, a single-cell suspension was prepared. Macrophages were cultured with the bacteria at an MOI (1:10) in DMEM. After 4 hrs of infection, extracellular bacteria were washed using 1× PBS. DMEM containing gentamycin was added to kill the remaining extracellular bacteria. Further, macrophage cells were lysed in 0.01% SDS and plated onto the 7H11-agar plates to determine the colony-forming unit (CFU). Western blotting was performed as described above to determine the protein levels.

### Immunofluorescence and confocal microscopy

Macrophage cells were cultured on a glass coverslip in a culture plate to investigate the localization of NFKB1 (P50) and RelA (P65). Cells were grown with varying concentrations of MoxR1 for 24 hrs. Furthermore, cells were washed with PBS and fixed in chilled methanol. 5% BSA was used for blocking non-specific sites. The following day cells were incubated with anti-mice αNFKB1, αpP65, and αTLR4 antibodies as recommended. Post-secondary antibody treatment cells were mounted onto glass slides using a Pro-long antifade mountant containing DAPI.

For colocalization of MoxR1 and TLR4, 20 µg/ml of MoxR1 was treated to RAW264.7 cells for 2 hrs, and cells were fixed and incubated with primary antibodies (αTLR4 and αMoxR1) overnight at 4℃. The signal was detected using Alexa fluor-conjugated secondary antibodies (A488 and A594).

### Homology modelling, docking, molecular dynamics simulations, and principal component analyses of MoxR1-TLR4 complex

We retrieved *Mycobacterial* MoxR1 (Q79FN7) protein sequence from the UniProtKB database. MoxR1 structure was predicted using i-Tasser and was checked for correct fold architecture by comparing respective Z-scores [27]. Before protein-protein docking of MoxR1 with TLR4, we checked the ProSA profile and Ramachandran plots to assess the correct conformation of side chains and tertiary architecture. The modelled MoxR1 was docked onto TLR4-dimer using ClusPro v2.0 software [28]. The top-scoring model was retained for use in further analysis. The docking protocol in ClusPro employs electrostatic, hydrophobic, and (Vander Waals + Electrostatic) interactions ranked by centres of clusters with low energy to define the native sites assuming to have wide free-energy attractors with the most significant number of results [29]. Molecular dynamics simulations of TLR4-dimer (TLR4)_2_) and TLR4-MoxR1 tetramers {(TLR4)_2_-(MoxR1)_2_} were carried under GROMOS54a7 all-atom force field of GROMACS 5.1 with periodic boundary conditions applied in all directions [30,31]. The SPC/E water model was used to solvate protein molecules at 1 nm from each box edge. The systems were maintained at 0.1 M NaCl electroneutrality by adding counter ions followed by energy minimization by the steepest-descent method. Subsequently, 1 ns NPT-NVT equilibration runs were performed along with positional restraints. The production MD (at 310K) runs of 50 ns for all systems were maintained by the Parrinello-Rahman barostat and Berendsen thermostat. At the same time, the Particle-Mesh-Ewald method was used to treat electrostatic contributions. Simulation data were analysed using in-built gromacs modules and PyMol. The PyMoL was used to generate images. The Principal Component Analysis (PCA) of TLR4-dimer and TLR4-MoxR1 tetramers was performed, as described earlier [25].

### The analysis of the MoxR1 for discovering LIR-motif

The iLIR tool was used to discover the conserved LIR motif in MoxR1 (https://ilir.warwick.ac.uk/lirpredict.php).

### Statistical analysis

One-way and two-way ANOVA with Tukey’s and Dunnett’s multiple comparisons were used to analyse the data. Three independent experiment readings were used to calculate means ± SD. P values≤0.05 were deemed significant. In order to analyse the data, GraphPad Prism 9 was used (San Diego, CA, USA).

## Results

### MoxR1 binds and activates TLR4 on the surface of macrophage cells

**Figure 1.**
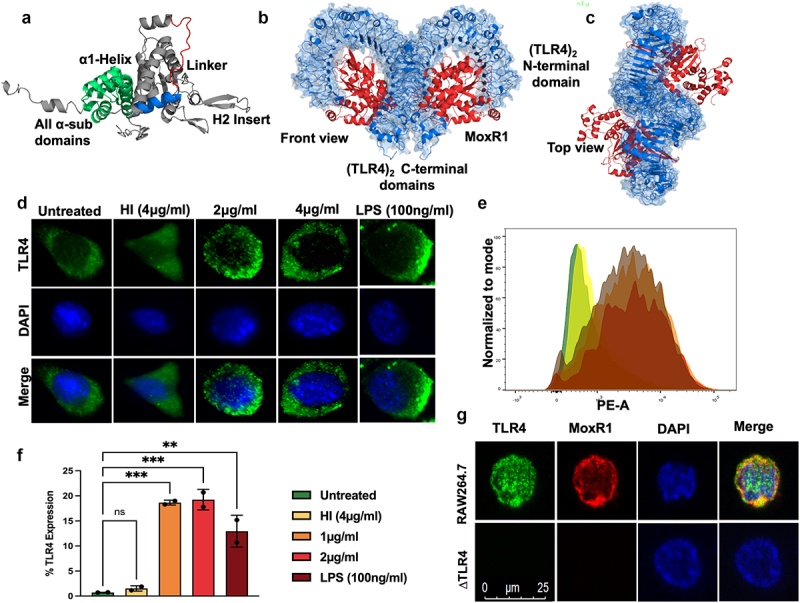
**MoxR1 induced the expression of TLR4 along with its organization in the plasma membrane**. (**a**) the modelled and simulated structure of MoxR1 shows its conserved domains and motifs. (**b**) Front view (**c**) Top View of (TLR4)_2_-(MoxR1)_2_ obtained through Clus-Pro protein docking. (**d**) Immunofluorescence images of TLR4 organization in the membrane. Increasing concentrations of MoxR1 were treated to RAW264.7 cells, and after 4 hrs of treatment, cells were used in immunofluorescence labelling. (**e**) FACS analysis showed enhanced expression of TLR4 in MoxR1-stimulated RAW264.7 cells. (**f**) Bar graph shows the quantitative representation of TLR4 expression in RAW264.7 cells. (**g**) Co-localization of MoxR1 and TLR4 in RAW264.7 cells stimulated with MoxR1. Two-way ANOVA was used for data analysis. Data are presented as means ±SD in comparison to the control. P value less than × 0.05, **0.01, ***0.001. All the graphs were prepared in GraphPad 9.0.

The crystal structure of *M. tb* MoxR1 is unknown yet; therefore, we searched for the closest homolog with a known structure. The *E. coli* RavA (Regulatory ATPase variant A), *Cytophaga hutchinsonii* putative ATPase, and *Rhodobacter capsulatus* BchI, one of the subunits of Mg^+2^ chelatase containing AAA+ (ATPase associated with various cellular activities) domain, are the three closest homologs to *M. tb* MoxR1 for which structures are known [32–35]. Using the structure of these MoxR1 homologs, we modelled and generated the structure of MoxR1 and observed that it contains a conserved ɑ1-Helix, a linker region, H2-insert, and all ɑ-subdomains (Figure 1a). Our multiple sequence analysis showed that MoxR1 comprises evolutionarily conserved essential motifs involved in ATP hydrolyses, such as Walker A and B motifs, sensor regions I and II, and arginine finger (**Figure S2A**).

We used protein-protein docking to elucidate the interaction of MoxR1 with TLR2 or TLR4 [36]. The molecular docking and simulation observations show that MoxR1 binds to the ligand recognition domain of TLR4 (Figures 1(b,c)). We used the top-scoring model of (TLR4)_2_(MoxR1)_2_ heterotetramer generated using ClusPro in molecular dynamics simulation studies to confirm our findings. The resultant trajectories were analysed for macroscopic parameters such as backbone RMSD (route mean square deviation) and Rg (gyration radius) of (TLR4)_2_ alone and in complex with (MoxR1)_2_. The interaction of CTD of TLR4 monomers (**Figure S2 B, C**, **and D**) acts as a crucial step in regulating the downstream cascade emanating from TLR4. Consequently, we studied the interaction of CTD in terms of variations in inter-CTD distance, H-bond number (HBN), and inter-CTD dihedral angle along the simulation trajectories. Following constructing free energy landscapes (FEL), FEL projected onto the inter-CTD distance vs. HBN, PCA (principal component analysis) of Cα atoms averaged throughout the entire trajectory was performed. The FEL projections displayed the single lowest free energy basin in simulations of (TLR4)2 alone (**Figure S2B**). The most stable and minimum energy requiring confirmations were further characterized by (TLR4)_2_ species consisting of 5–7 H-bonds and a minimum distance of ~1.5 nm between the CTD (**Figure S2 B, C, and D**). However, in the bound state with MoxR1, the FEL showed a large free energy basin corresponding to (TLR4)_2_ structures with a maximum of 12 H-bonds and a minimum distance of ~0.8 nm between the CTD (**Figure S2 B, C, and D**). Interestingly, minimal Cα fluctuations were observed in (TLR4)_2_ architecture upon binding with the MoxR1. The porcupine analysis revealed that the binding of MoxR1 reduced the overall fluctuations in the (TLR4)_2_ architecture and brought the CTD into proximity mechanistically by favouring the formation of a stable H-bond network between the CTD (**Figure S2D**).

We used *in-vitro* experiments to confirm our computational analysis using RAW264.7 cells and endotoxin-free MoxR1 protein. Analysis of immunofluorescent images shows that MoxR1 activates the expression of TLR4 and potentiates its targeting to the macrophage cell surface, which is a prerequisite for initiating an activated downstream signalling cascade (Figure 1d). To further strengthen our findings, we employed flow cytometric analysis using PE-tagged anti-TLR4 antibody and revealed that MoxR1 induced the increased expression of TLR4 in RAW264.7 cells in a concentration-dependent manner (Figure 1(e,f)) Additionally, we used a laser scanning confocal microscope to study colocalization to demonstrate the interaction between MoxR1 and TLR4 at the plasma membrane. The colocalization of MoxR1 crosslinked at the macrophage cell surface with TLR4 was analysed using Alexa fluor (488 and 594) linked secondary antibody. We observed MoxR1 and TLR4 colocalization in macrophage cells, suggesting their interaction (Figure 1g). Our findings show that the MoxR1 is a TLR4-specific ligand required for inducing TLR4 expression.

### MoxR1 activates TLR4 to induce heightened secretion of proinflammatory cytokines

**Figure 2.**
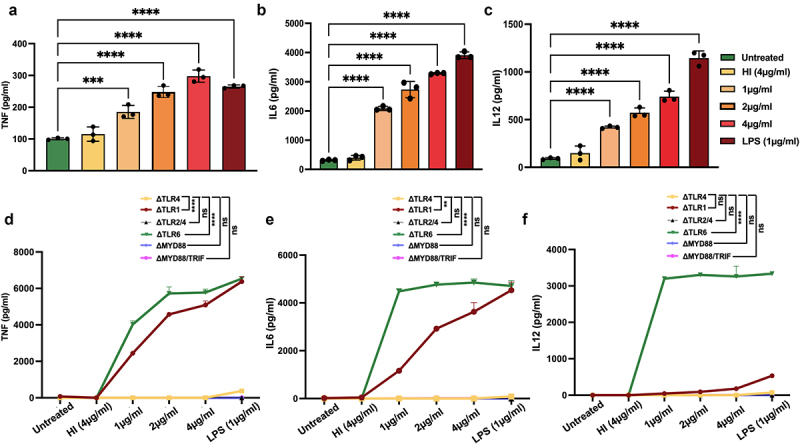
**MoxR1 induced the enhanced secretion of inflammatory cytokines**. Level of (**a**) TNF (**b**) IL6 (**c**) IL12 produced when RAW264.7 cells were stimulated with the increasing amount of MoxR1. Levels of (**d**) TNF, (**e**) IL6, and (**f**) IL12 were produced when TLR knockout cell lines were treated with MoxR1. Sandwich ELISA was used for measuring cytokines. LPS stimulation was used as positive control. The mean from three independent experiments was used to calculate the standard deviation. Data are presented as means ±SD in comparison to the control. P value less than × 0.05, **0.01, ***0.001. All the graphs were prepared in GraphPad 9.0.

The interaction of MoxR1 with the ligand recognition domain of TLR4 prompted us to study its role in regulating cytokine secretion. The cell culture supernatant of RAW264.7 cells was collected after treatment with MoxR1 protein, and levels of pro-and anti-inflammatory cytokines were quantified utilizing sandwich ELISA. The results demonstrated that MoxR1 elicited proinflammatory cytokine production in a concentration-dependent manner, including TNF, IL6, and IL12. (Figures 2(a–c)). Notably, no change was detected in the expression of IL10 (**data not shown**). TLRs, along with their downstream adapters MYD88 and TRIF, are known to have a role in cytokines production by *M. tb* antigens. Thus, we employed knockout cell lines of various TLRs such as ΔTLR1, ΔTLR2/4, ΔTLR4, ΔTLR6, ΔTRIF, and ΔMYD88/TRIF decipher the innate immune receptor involved in producing anti-TB cytokines, including TNF, IL6, and IL12 using ELISA. We found that MoxR1 stimulated ΔTLR1 and ΔTLR6 macrophage cells to produce proinflammatory cytokines, whereas no significant amounts of cytokines were produced by ΔTLR4, ΔTLR2/4, ΔTRIF, and ΔMYD88/TRIF cells (Figure 2(d–f)) Our findings suggest that MoxR1 is a potent activator of proinflammatory cytokine secretion utilizing the TLR4 and downstream adapter protein MYD88.

### MoxR1 activates canonical NFKB and mitogen-activated protein kinase pathways for sustained production and secretion of proinflammatory cytokines

**Figure 3.**
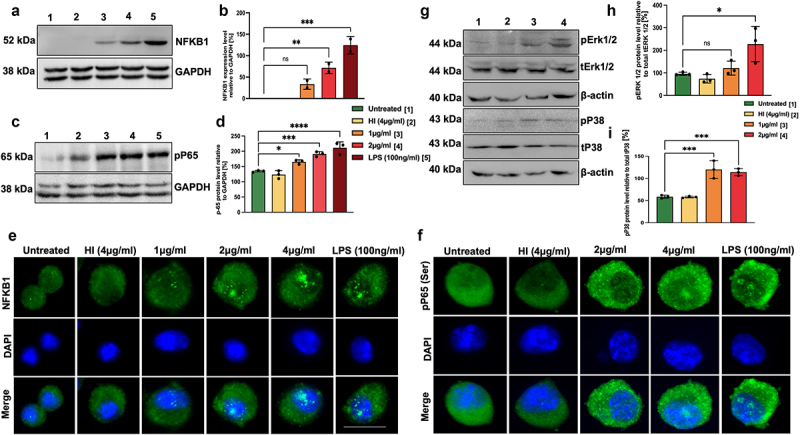
**Proinflammatory cytokine secretion by MoxR1 depends on the activation of NFKB and MAPK signalling pathways**. (**a**) Western blot analysis of enhanced NFKB (P50) expression with increasing concentration of MoxR1 treated RAW264.7 cells. (**b**) Level of NFKB1 was quantitated and normalized to GAPDH [%]. (**c**) Western blot depicting the phosphorylated pP65 subunit of NFKB signalling pathway after MoxR1 treatment to RAW264.7 cells for 24 hr. (**d**) RelA (pP65) level was measured and normalized to GAPDH [%]. (**e**) Immunofluorescence images show enhanced localization of NFKB (P50) subunit in the nucleus with increasing concentration of MoxR1 treatment to murine macrophage cells (RAW264.7) (**f**) Immunofluorescence images showing nuclear localization of RelA (pP65) subunit with increasing concentration of MoxR1 treatment to macrophage cells (RAW264.7). The scale bar represents 10 µm. (**g**) Western blot analysis of pERK, tERK, pP38, tP38 in MoxR1 treated RAW264.7 cell line. (**h**) pERK1/2 was quantitated and normalized to tERK1/2 [%]. (I) pP38 was measured and normalized to tP38 [%]. The mean from two or three independent experiments was used to calculate the standard deviation. Data are presented as means ±SD in comparison to the control. P value less than × 0.05, **0.01, ***0.001. All the graphs were prepared in GraphPad 9.0.

TLR2 or TLR4 mediated recruitment of adapter proteins MYD88 or TRIF activates downstream signalling cascades such as NFKB and MAPK p38/ERK1/2 signalling for producing proinflammatory cytokines to control *M. tb* infection [7,26,37]. We analysed the activation status of NFKB and MAPK signalling pathways by detecting the activatory phosphorylation using phosphorylation-specific antibodies. We observed that MoxR1 activated the increased expression of P50 and induced the activatory phosphorylation of P65 (Ser536), ERK1/2 (Thr202/Tyr204), and p38 (Thr180/Tyr182) (Figure 3(a–I)) The nuclear localization of NFKB is essential for producing proinflammatory cytokines, and it is a hallmark of activated NFKB signalling [38]. MoxR1 induced targeting of P50 and pP65 into the nucleus, as shown by the increased foci formation (Figure 3(e,f) . The nuclear foci formation is a marker of activated transcription, thus suggesting that MoxR1 is a potent activator of NFKB and MAPK signalling pathways required for increased secretion of proinflammatory cytokines.

### MoxR1 is a potent autophagy inhibitor of RAW264.7 macrophages

**Figure 4.**
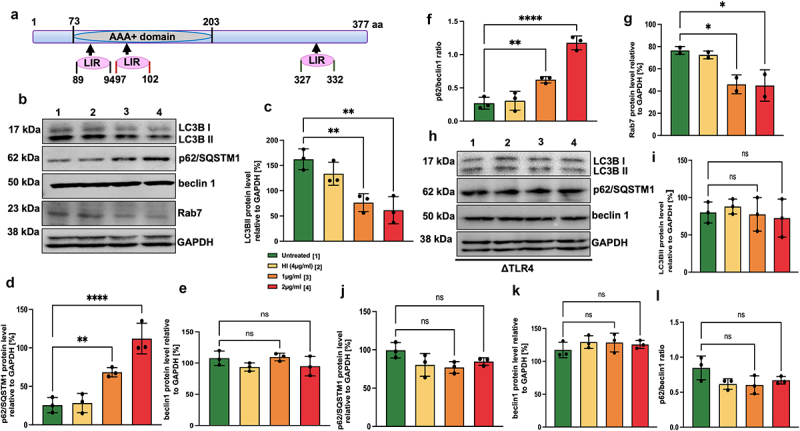
**Inhibition of autophagy by MoxR1 is dependent on TLR4**. (**a**) Pictorial representation of domains of MoxR1 and positions of putative LC3-interacting region (LIR) motifs shown with an arrowhead. (**b**) Western blot analysis representing the expression of autophagy markers LC3II, P62, beclin1, and Rab7 in MoxR1 treated RAW264.7 cells. (**c**) LC3BII (**d**) p62/SQSTM1 (**e**) beclin 1 (**f**) P62/beclin1 ratio (**g**) Rab7 was measured and normalized to GAPDH [%]. (**h**) Western blot analysis representing the expression of autophagy markers LC3BII, P62, and beclin1 in MoxR1 treated ΔTLR4 cells. (**i**) LC3BII (**j**) p62/SQSTM1 (**k**) beclin 1 (**l**) P62/beclin1 ratio was quantitated and normalized to GAPDH [%] in MoxR1 treated ΔTLR4 cells. The mean from two or three independent experiments was used to calculate the standard deviation. Data are presented as means ±SD in comparison to the control. P value less than × 0.05, **0.01, ***0.001. All the graphs were prepared in GraphPad 9.0.

**Figure 5.**
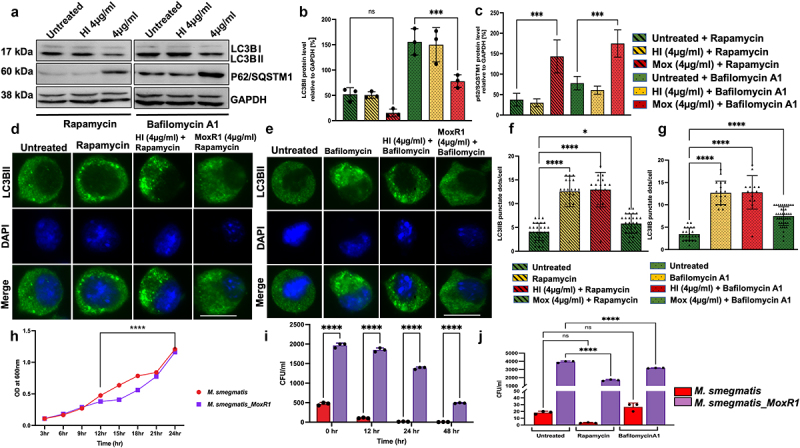
**MoxR1 downregulated the autophagy flux by inhibiting the autophagy initiation**. (**a**) Western blot was used to analyse the expression of autophagy markers such as LC3BII and SQSTM1 in macrophages treated with MoxR1 and rapamycin (200 nm) or bafilomycin A1 (50 nm). (**b**) LC3BII was measured and normalized to GAPDH [%]. (**c**) p62/SQSTM1 was quantitated and normalized to GAPDH [%]. (**d**) Immunofluorescence images showing the LC3BII puncta foci produced in MoxR1 and/or rapamycin-treated RAW264.7 cells. (**e**) Immunofluorescence images showing the LC3BII puncta foci produced in MoxR1 and/or bafilomycin A1 stimulated RAW264.7 cells. The nucleus was stained using DAPI. (**f and g**) the bar graph represents the average number of LC3BII puncta foci generated when RAW264.7 was treated with MoxR1, rapamycin, and bafilomycinA1. (**H**) Growth curve analysis of *M. smegmatis* vector alone, and *M. smegmatis* expressing MoxR1 (**i**) Intracellular survival of vector alone, and *M. smegmatis* expressing MoxR1 after the infection (MOI 1:10) in RAW264.7 cells. After infection, cells were lysed in 0.01% SDS containing 1×PBS, and dilutions were plated onto a 7H11 agar plate supplemented with OADC. On the fourth day, colonies on agar plates were counted (**j**). Growth of *M. smegmatis* vector alone and MoxR1 expressing *M. smegmatis* was analysed in the presence of bafilomycin A1 and rapamycin. Post-infection (MOI 1:10), RAW264.7 cells were stimulated with either rapamycin (200 nm) or bafilomycin (50 nm). Cells were lysed, and CFU was determined on the fourth day of plating. The mean from two or three independent experiments was used to calculate the standard deviation. Data are presented as means ±SD in comparison to the control. P value less than × 0.05, **0.01, ***0.001. All the graphs were prepared in GraphPad 9.0.

Our *in-silico* analysis identified that MoxR1 comprises conserved LC3-interacting region (LIR) motif (89–94 aa., WxxL; 97–102 aa., WxxL; and 327–332 aa., LTYDAL) (Figure 4a). LC3 motif-containing proteins are known regulators of autophagy initiation, phagophore formation, maturation, and fusion to the lysosome to form degradative autolysosome [39]. Moreover, as TLR4 is known to act as a sensor for autophagy modulation [40], these findings prompted us to decipher the role of MoxR1 in autophagy regulation. The reduction in the conversion of LC3BI to LC3BII, a marker protein used to examine autophagy regulation, after MoxR1 treatment of macrophages demonstrated effective autophagy inhibition (Figures 4(b,c)). Additionally, our Western blot analysis further showed the reduced utilization of autophagy substrate SQSTM1/P62, confirming the role of MoxR1 in autophagy inhibition (Figures 4(b,d)). Moreover, we also observed a high P62/beclin 1 ratio suggestive of a MoxR1-dependent efficient inhibition of autophagic flux (Figure 4(b–f). The Rab family of small GTPases are known regulators of endosome and phagophore maturation. Notably, we observed reduced Rab7 levels in MoxR1-treated macrophages, indicating that MoxR1 is a potent autophagy inhibitor (Figure 4(b,g)). As earlier demonstrated, MoxR1 is a potent TLR4 agonist, and we, therefore, sought to reveal the role of TLR4 in autophagy regulation. Interestingly, we observed no change in protein levels of classical autophagy markers in ∆TLR4 macrophage cells treated with MoxR1 (Figure 4(h–l)). These findings suggest that MoxR1 is a potent inhibitor of autophagy mediated by TLR4. Further, to determine the mechanistic details of autophagy inhibition by MoxR1, RAW264.7 cells were treated with MoxR1 (4 µg/ml) in the presence of autophagy-modulating drugs such as rapamycin and bafilomycin A1, which are used to determine the autophagy flux. While bafilomycin A1 effectively prevents the fusion of the autophagosome to the lysosome, which results in the creation of autolysosomes, rapamycin stimulates autophagy by blocking the activity of autophagy master regulator MTOR (**Figure S2E)**. Intriguingly, our results show that MoxR1 is a potent inhibitor of autophagy flux, as shown by the reduced level of LC3BII membranous structures when combined with autophagy-regulating drugs rapamycin and bafilomycin A1, which is an indicator of autophagy inhibition. Moreover, we also observed an increased level of P62 accumulation, an autophagy substrate, upon treatment with MoxR1 (Figures 5(a–c)). Furthermore, to corroborate our results, we investigated the autophagy-regulating function of MoxR1 by examining the formation of LC3BII puncta with rapamycin and bafilomycin A1. We demonstrate that MoxR1 inhibits the formation of LC3BII punctate foci, as revealed by the reduced number of LC3BII foci in the presence of pharmacological drugs rapamycin and bafilomycin (Figure 5(d–g)). Our observations imply that MoxR1 inhibits autophagy initiation to decrease the autophagy flux. To further validate our findings, we used *M. smeg* as a surrogate model most commonly used to decipher the function of *M. tb* genes/proteins. We observed a reduced growth rate of *M. smeg* containing *moxR1* compared to the negative control *M. smeg* comprising vector alone, suggestive of enhanced virulence of slow-growing pathogenic variants (Figure 5h). Furthermore, we analysed the role of MoxR1 in the survival of *M. smeg* within macrophages by infection at an MOI (1:10). We infected RAW264.7 cells with *M. smeg* containing either *moxR1* or vector alone. The macrophage survival was analysed after 24 and 48 hrs and represented as a CFU. We observed that *M. smeg* expressing MoxR1 showed robust survival within macrophages compared to vector alone containing cells (Figure 5i). This observation suggests that *M. smeg* expressing MoxR1 inhibits clearance of recombinant bacteria – a virulence potential acquired by integrating MoxR1 in its genome. We also find that *M. smeg* expressing MoxR1 shows robust survival inside macrophage cells, as evidenced by the increased CFU/ml of *M. smeg* cells harbouring moxR1 compared to the control cells containing vector alone when cultured with autophagy modulating agents rapamycin and bafilomycin A1 (Figure 5j) . Our findings suggest that *M. smeg* expressing MoxR1 inhibits the innate defences of RAW264.7 cells for increased survival even in the presence of autophagy inducer and inhibitor and despite exhibiting slower growth kinetics when compared to the control *M. smeg* cells co – ntaining vector alone.

### MoxR1 inhibited autophagic flux *via* activation of PI3K-AKT-MTOR-ULK1 signalling cascade using innate surface receptor TLR4

**Figure 6.**
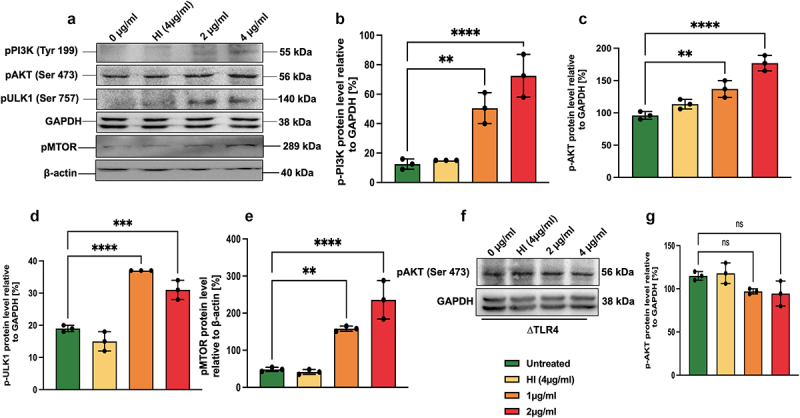
**Inhibition of autophagy by MoxR1 is mediated through activation of the PI3K-AKT-MTOR-ULK1 signalling axis**. (**a**) Western blot analysis shows activated phospho pPI3K, pAKT, pMTOR, and pULK1 kinases in MoxR1 (2 and 4 µg/ml) treated RAW264.7 cells. The MoxR1 concentrations used in treating macrophages and the molecular weight of the activated/inhibited phospho enzymes in response to MoxR1 and phospho antibodies are marked in the figure. (**b, c, d, and e**) *p*-PI3k, p-AKT, p-ULK1, and pMTOR protein levels were quantified and normalized to GAPDH or β-actin [%]. (**f**) Western blot analysis showing the expression of p-AKT in MoxR1 treated to ΔTLR4 cells. (**g**) the p-AKT level was measured and normalized to GAPDH [%]. The mean from two or three independent experiments was used to calculate the standard deviation. Data are presented as means ±SD in comparison to the control. *P* value less than × 0.05, **0.01, ***0.001. All the graphs were prepared in GraphPad 9.0.

We have shown in earlier results that MoxR1 is an efficient autophagy inhibitor in RAW264.7 cells. Moreover, MoxR1 also exhibited autophagy-inhibiting properties even in the presence of potent autophagy-regulating drugs rapamycin and bafilomycin A1, suggesting that it regulates autophagy in the initial steps. Activation of the PI3K-AKT-MTOR signalling cascade inhibits autophagy and apoptosis. Western blot analysis revealed that MoxR1 activated the kinases PI3K, AKT, and MTORC1 by inducing the phosphorylation required to activate them. Notably, MoxR1 treatment to RAW264.7 cells inhibited the kinase ULK1, which is required for autophagy initiation and phagophore biogenesis (Figure 6(a–e)). MTOR is a master regulator of autophagy; its activation leads to the suppression of autophagy. The activity of ULK1 is regulated by activatory and inhibitory phosphorylation, in which AMPK-mediated phosphorylation activates ULK1 while MTOR-mediated phosphorylation inhibits its activity. Together, our results suggest that MoxR1-mediated activation of PI3K-AKT-MTOR effectively inhibits autophagy initiation and autophagic flux. Furthermore, we also confirmed that MoxR1-mediated inhibition of autophagy and activation of PI3K-AKT-MTOR is TLR4 dependent, as evidenced by the similar phosphorylation of AKT in ΔTLR4 macrophages (Figure 6(f,g)),g.

### MoxR1 represses apoptosis *via* inhibiting protooncogene c-FOS and MAPK JNK1/2

**Figure 7.**
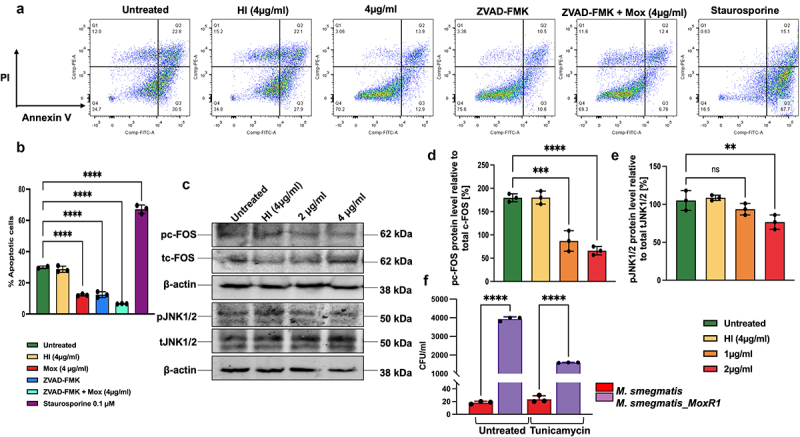
**MoxR1 inhibited apoptosis by inhibiting the transcription factors pc-FOS and p-JNK1/2 in RAW264.7 cells. (a)** FACS analysis of the different stages that is, early and late apoptosis in MoxR1 treated murine macrophages. Untreated, Heat inactivated, and ZVAD-FMK was used as the negative control, whereas Staurosporine stimulation was used as the positive control. **(b)** The bar graph shows the early apoptotic cells in different samples, as marked in the figure. **(c)** Western blot analysis shows the expression of pc-FOS, tc-FOS, pJNK1/2, tJNK1/2, and β-actin. **(d and e)** Bar graph showing the protein level of phosphoproteins normalized to the total proteins [%]. **(f)** CFU/ml was used to analyze the intracellular survival of M. smegmatis (vector alone and recombinant) infected with macrophage cells. Post-infection cells were treated with tunicamycin for 4 hrs, and after 24hrs, cells were lysed and plated onto a 7H11 agar plate supplemented with OADC. The mean from two or three independent experiments was used to calculate the standard deviation. Data are presented as means ±SD in comparison to the control. P value less than *0.05, **0.01, ***0.001. All the graphs were prepared in GraphPad 9.0.

Activation of AKT is involved in the positive regulation of MDM2, a ubiquitin ligase that determines the protein level of pro-apoptotic tumour suppressor TP53, a master apoptosis regulator [41]. The activation of pro-survival kinases such as PI3K, AKT, and MTOR in the presence of MoxR1 and its potent inhibitory effect on autophagic flux/autophagy initiation prompted us to unveil its role in apoptosis regulation. The FACS results indicated that MoxR1 significantly inhibited apoptosis, and the inhibition was more pronounced when combined with Z-VADFMK, an inhibitor of caspase-dependent apoptosis (Figures 7(a,b)). Earlier studies have shown that the activation of JNK and cFOS induce apoptosis [42]. The Western blot results revealed that MoxR1 efficiently inhibited the activation of JNK1/2 and cFOS (Figures 7(c–e)).

Induction of the UPR pathway leads to increased apoptosis, and it is one of the mechanisms to clear the non-pathogenic mycobacteria. We employed recombinant *M. smeg* containing *moxR1* to infect the RAW264.7 cells and studied the protein levels of the endoplasmic reticulum-associated unfolded protein response (UPR) pathway. The results showed that the *M. smeg* expressing MoxR1 might also be inhibiting the UPR pathway, as demonstrated by a slightly lower level of UPR markers such as BIP, IRE1α, and PDI (**Figure S2F**). Moreover, to study the mechanistic role of MoxR1 in inhibiting the ER-mediated stress such as the UPR pathway, vector and MoxR1 over-expressing *M. smeg* cells were infected to RAW264.7 cells in the presence of a potent inducer of UPR pathway, tunicamycin, and the survival was analysed using CFU assay. Interestingly, *M. smeg* expressing MoxR1 showed robust growth in the presence of UPR-inducing drug tunicamycin compared to control *M. smeg* comprising vector alone, as evidenced by the increased CFU/ml (Figure 7f). Tunicamycin induces ER-associated UPR by inhibiting the N-glycosylation of proteins [43]. These findings imply that MoxR1 of *M. tb* is an efficient inhibitor of host cell apoptosis *via* inhibiting the activation of the UPR pathway for robust intracellular survival within macrophage cells.

### MoxR1 efficiently inhibited the production of enzymes involved in oxidative phosphorylation for inducing metabolic repurposing

**Figure 8.**
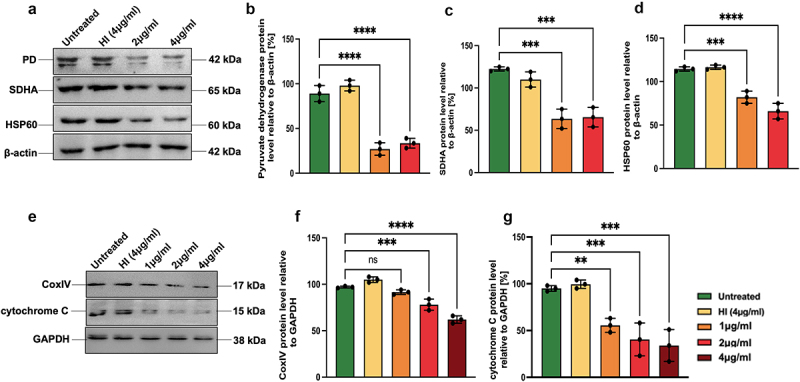
**MoxR1 induced robust metabolic repurposing by inhibiting the mitochondrial bioenergetics. (a)** the expression of the citric acid cycle and electron transport chain proteins, including succinate Dehydrogenase (SDHA), pyruvate dehydrogenase (PD), and HSP60, was analysed using western blot analysis. (**b, c, and d**) Bar graph showing the protein quantitation normalized with β-actin. (**e**) Western blot shows the expression of enzymes CoxIV and CytC in the MoxR1 treated RAW264.7 cells after 24 hrs of treatment. (**f and g**) Bar graph showing the quantitative analysis of proteins CoxIV and CytC normalized to β-actin [%]. The mean from two or three independent experiments was used to calculate the standard deviation. Data are presented as means ±SD in comparison to the control. P value less than × 0.05, **0.01, ***0.001. All the graphs were prepared in GraphPad 9.0.

Many bacterial pathogens induce metabolic repurposing or Warburg phenotype to rewire the metabolic intermediates for their benefit, such as *M. tb* and *L. pneumophila* [44,45]. However, the candidate effector proteins that induce metabolic reprogramming are emerging now. In our earlier study, we demonstrated that RipA of *M. tb* efficiently inhibited the oxidative phosphorylation pathway enzyme; Succinate dehydrogenase (SDHA), Pyruvate dehydrogenase (PD), CoxIV, Cytochrome C (CytC), and HSP60 [26]. Therefore, we explored the role of MoxR1 in inducing metabolic reprogramming in RAW264.7 cells. The results demonstrated that MoxR1 inhibits the production of the citric acid cycle and electron transport chain enzymes, including PD, SDHA, CytC, and CoxIV (Figures 8(a–g)). Surprisingly, we observed a reduced level of HSP60 (Figure 8G), a mitochondrial chaperone, and an essential protein that plays a critical role in various stress conditions for the survival of the host cells [46]. Therefore, our findings suggest that MoxR1 inhibits the production of enzymes involved in oxidative phosphorylation, probably to rewire the citric acid cycle intermediates for their utilization. The results also suggest that MoxR1 might activate glycolysis to rapidly produce ATP for efficient energy utilization. Furthermore, we found that the downregulation of ETC enzymes was TLR4 dependent as there was no change in the expression in TLR4 knockout macrophage cells.

## Discussion

Over the millennia, *M. tb* and humans have evolved compelling crosstalk between their effector proteins in the fight for survival. Hence, it becomes pivotal to delineate the role of effector proteins in manipulating the host survival strategies. Pattern Recognition Receptors (PRRs) play an essential role in sensing the presence of pathogenic effectors surrounding them and initiate a series of signalling pathways that could ultimately result in pathogen clearance [47]. In this study, we have characterized one of the evolutionary conserved MoxR family proteins of *M. tb*. Our findings unveiled that MoxR1 is a potent activator of TLR4, NFKB, and MAPK signalling cascades required to produce the increased amount of anti-TB cytokines TNF, IL6, and IL12. MoxR1 interacts and activates the TLR4 and its membrane localization, a prerequisite for its activation. It is worth mentioning that *M. tb* secreted or cell surface proteins usually interact with TLR2 or TLR4 [8]. The interaction of *M. tb* proteins with these receptors either activates or represses the secretion of pro- or anti-inflammatory cytokines to modulate host-pathogen interaction. The production and secretion of cytokine effectors are then mediated by activation or repression of NFKB and MAPK signalling [48]. The host is believed to produce proinflammatory cytokines to control the infection. In contrast, bacterial pathogens, including the intracellular *M. tb*, using its virulence factors to dampen the elicitation of host protective anti-TB inflammatory cytokines and induce the secretion of anti-inflammatory cytokines to subdue host immunity [26,49]. Interestingly, it is also reported that *M. tb* utilizes its virulence factors to heighten the secretion of proinflammatory cytokines such as TNF to induce host tissue damage that ultimately culminates in its dissemination [50]. We demonstrate that MoxR1 required surface TLR4 and downstream adapter MYD88 for secreting proinflammatory cytokines, supported by the observation that TLR4 and MYD88 knockout mouse macrophage cells cannot secrete these cytokines. The findings support our hypothesis that MoxR1 is a specific and potent TLR4 agonist that interacts with the ligand recognition domain of TLR4, essentially requiring to recruit MYD88 to activate NFKB and MAPK signalling.

Besides cytokine signalling, *M. tb* inhibits cell death pathways for successful intracellular replication [51,52]. It was intriguing to observe that mycobacterial proteins encompass eukaryotic-like motifs that possibly modulate host cell signalling. *In-silico* analysis revealed an LIR motif in the MoxR1 sequence that executes essential functions during autophagy regulation in eukaryotes [53,54]. It has been shown that LIR-motif-containing proteins act as autophagy receptors to connect the ATG8-family proteins to the expanding phagophore membrane and help in phagophore biogenesis and fusion of autophagosome to the lysosome to form autolysosome for degradation of cargo materials including bacterial and viral pathogens [54–56]. MoxR1 inhibited autophagy and the production of RAB-family small GTPase protein RAB7, which is essential for phagophore maturation [57,58]. Mechanistic exploration reveals that MoxR1 induces activatory phosphorylation of PI3K, AKT, and MTOR (the master regulator of autophagy). The activation of MTOR culminates in inducing inhibitory phosphorylation of ULK1, which is essentially required to inhibit autophagy initiation. These findings implicate that MoxR1 is a potent inhibitor of autophagy initiation, which depends on the innate immune receptor TLR4 and the downstream master autophagy regulator MTORC1 together with ULK1, whose activity is required for efficient autophagy initiation.

Programmed cell death pathways such as autophagy and apoptosis provide immunity against diverse viral and bacterial pathogens. Both these processes are critical in providing immunity against intracellular *M. tb* and controlling its replication, survival, and virulence within the host. It is also reported that autophagy and apoptosis are linked pathways that may be regulated positively or negatively [7,26,59,60]. We demonstrate that MoxR1 inhibits apoptosis by inhibiting the pro-apoptotic MAPK JNK1/2 and transcription factor cFOS. Moreover, recombinant *M. smeg* expressing MoxR1 demonstrate that MoxR1 downregulates ER-stress pathways to possibly limit the apoptotic mode of cell death. Our study shows that MoxR1-mediated inhibition of apoptosis and autophagy aids in survival, as shown by the enhanced intracellular replication by MoxR1 expressing *M. smeg* in macrophage cells. Mycobacteria employ various effectors to inhibit autophagy and apoptosis for their survival. PPE51, PE_PGRS20, PE_PGRS47, and PE6 are some of the effectors shown to enhance the intracellular survival of *M. tb* by inhibiting autophagy [7,61–63].

Intracellular pathogens such as *L. pneumophila* and *M. tb* are known to exploit host metabolic intermediates for their benefit [64,65]. The role of effector proteins that induce metabolic reprogramming or metabolic repurposing remains relatively unexplored. Earlier studies have shown that *M. tb* infection induces robust metabolic reprogramming for utilizing citric acid cycle intermediates for its benefit [66,67]. Here, we show that MoxR1 induces robust metabolic reprogramming by inhibiting the production of enzymes involved in the citric acid cycle and oxidative phosphorylation. MoxR1 inhibits the production of PD, SDHA, CytC, and CoxIV to suppress oxidative phosphorylation. We speculate that MoxR1-mediated inhibition of oxidative phosphorylation provides the opportunity for *M. tb* to use citric acid cycle intermediates for its metabolism. It is also safely speculated that *M. tb*-induced inhibition of oxidative phosphorylation alternatively activates glycolysis for ATP production with high kinetics to replenish the citric acid cycle for efficient production of intermediate metabolites.

**Figure 9.**
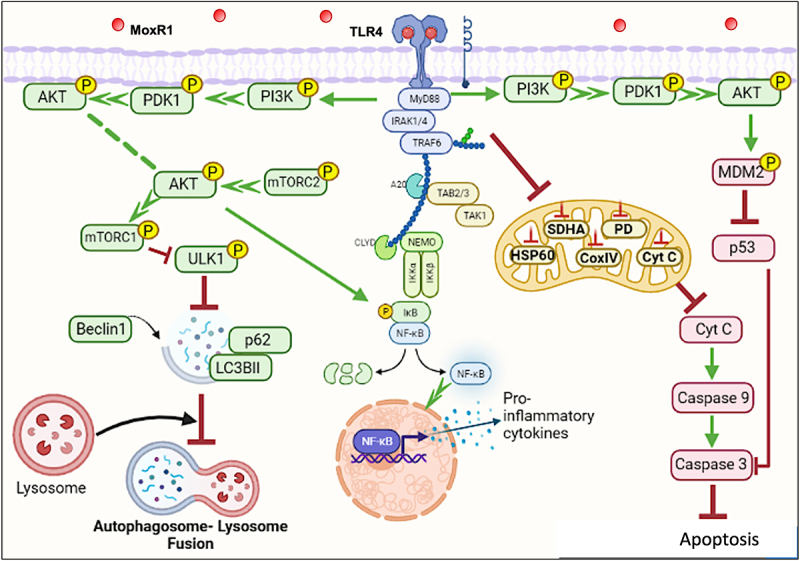
**MoxR1 is a potent TLR4 agonist that activates downstream NFKB and MAPK signalling cascades to suppress autophagy and apoptosis**. MoxR1 interacted with TLR4 to activate the downstream signalling cascades (NFKB and MAPK) by recruiting MYD88. The enhanced production of proinflammatory cytokines by Moxr1 depended on the activation of NFKB and MAPK signalling pathways. It inhibited autophagy, as evidenced by the protein levels of the classical autophagy markers. MoxR1 activated the pro-survival signalling cascade PI3k-AKT-MTOR to inhibit autophagy initiation. Moreover, MoxR1 also repressed MAPK JNK1/2 and cFOS to inhibit apoptosis, a crucial innate defence mechanism used to control the replication of pathogens. Notably, MoxR1 also induced robust metabolic reprogramming for rewiring the citric acid cycle intermediates for its benefit. Intracellular pathogens, including *L. pneumophila* and *M. tb*, inhibit mitochondrial oxidative phosphorylation for activating glycolysis-mediated production of ATP so that pathogen can exploit citric acid cycle intermediates for its metabolism. Our findings suggest that MoxR1 is a moonlighting protein of *M. tb* that the pathogen exploits to dampen host-directed defences for successful survival and virulence.

Our findings suggest that MoxR1 is a moonlighting protein that uses a multipronged approach to dampen host-directed immunity for efficient replication, survival, and pathogenesis (Figure 9). Understanding these proteins better by exploring the multipronged strategies to overcome host defences could unravel novel strategies for controlling tuberculosis. Moreover, modulation of autophagy and apoptosis by MoxR1 ligands can be explored as host-directed therapies for complementing the anti-tuberculous therapies.

## Supplementary Material

Supplemental Material

## Data Availability

Data available within the article or its supplementary materials
