# Supplementary material for: Mycobacterium tuberculosis protein MoxR1 enhances virulence by inhibiting host cell death pathways and disrupting cellular bioenergetics
Source: Virulence. 2023 Feb 26;14(1):2180230. doi: 10.1080/21505594.2023.2180230 (PMC9980616; doi:10.1080/21505594.2023.2180230)
Supplement: Supplemental Material [file KVIR_A_2180230_SM4284.zip › 2180230_-_Supplement/5. Table S1.docx]

Table S1. Bacterial strains and plasmid used in this study

| Strains and plasmids | Description | Source/Ref |
| --- | --- | --- |
| Strains |  |  |
| *E. coli* |  |  |
| ClearColi BL21(DE3) | *F– ompT hsdSB (rB- mB-) gal dcm lon λ (DE3 [lacI lacUV5-T7 gene 1 ind1 sam7 nin5]) msbA148 ΔgutQ ΔkdsD ΔlpxLΔlpxMΔpagPΔlpxP ΔeptA* | Biosearch Technologies |
| DH5α | *F^-^ [ϕ80dΔlacZM15] Δ(lacZYA-argF) U169 deoR recA1 endA1 hsd R17 glnV44 thi-1 gyrA96 relA1* | Gibco-BRL |
| *Mycobacterium* |  |  |
| *M. smegmatis* mc^2^155 | *ept-1, efficient plasmid transformation mutant* | [1] |
| Plasmids |  |  |
| pET28a | Km^r^, His tag protein expression vector | Novagen |
| pETMoxR1 | Km^r^, a fragment containing entire *MoxR1* coding region cloned in pET28a | This study |
| pST2K | Km^r^, a *M. smegmatis* dual constitutive expression vector containing His tag in MCS-1 and Flag tag protein in MCS-2 | [2] |
| p2KMoxR1 | Km^r^, a fragment containing entire *MoxR1 c*oding region cloned in MCS-1 in pST2K | This study |
|  |  |  |
|  |  |  |

1. Snapper, S.B., et al., *Isolation and characterization of efficient plasmid transformation mutants of Mycobacterium smegmatis.* Mol Microbiol, 1990. **4**(11): p. 1911-9.

2. Parikh, A., et al., *Development of a new generation of vectors for gene expression, gene replacement, and protein-protein interaction studies in mycobacteria.* Appl Environ Microbiol, 2013. **79**(5): p. 1718-29.
