# Supplementary material for: Mycobacterium tuberculosis protein MoxR1 enhances virulence by inhibiting host cell death pathways and disrupting cellular bioenergetics
Source: Virulence. 2023 Feb 26;14(1):2180230. doi: 10.1080/21505594.2023.2180230 (PMC9980616; doi:10.1080/21505594.2023.2180230)
Supplement: Supplemental Material [file KVIR_A_2180230_SM4284.zip › 2180230_-_Supplement/6. Table S2.docx]

Table S2. Primers used in this study.

| **Primer name** | **Nucleotide sequence (5’ 3’)** | **Comment** |
| --- | --- | --- |
| **Gene cloning** |  |  |
| pETMoxR1 F | ATGGATCCATGACATCAGCAGGTGGGTTC | Cloning of *MoxR1* into pET28a |
| pETMoxR1R | ATCTCGAGTCACCGGCCGCTCGCCGCGGC |  |
| p2KMoxR1F | ATGGATCCATGACATCAGCAGGTGGGTTC | Cloning of *MoxR1* into pST2K |
| p2KMoxRI R | AATCTAGACCGGCCGCTCGCCGCGGCCGC |  |
