# Supplementary figures and images for: Mycobacterium tuberculosis protein MoxR1 enhances virulence by inhibiting host cell death pathways and disrupting cellular bioenergetics
Source: Virulence. 2023 Feb 26;14(1):2180230. doi: 10.1080/21505594.2023.2180230 (PMC9980616; doi:10.1080/21505594.2023.2180230)

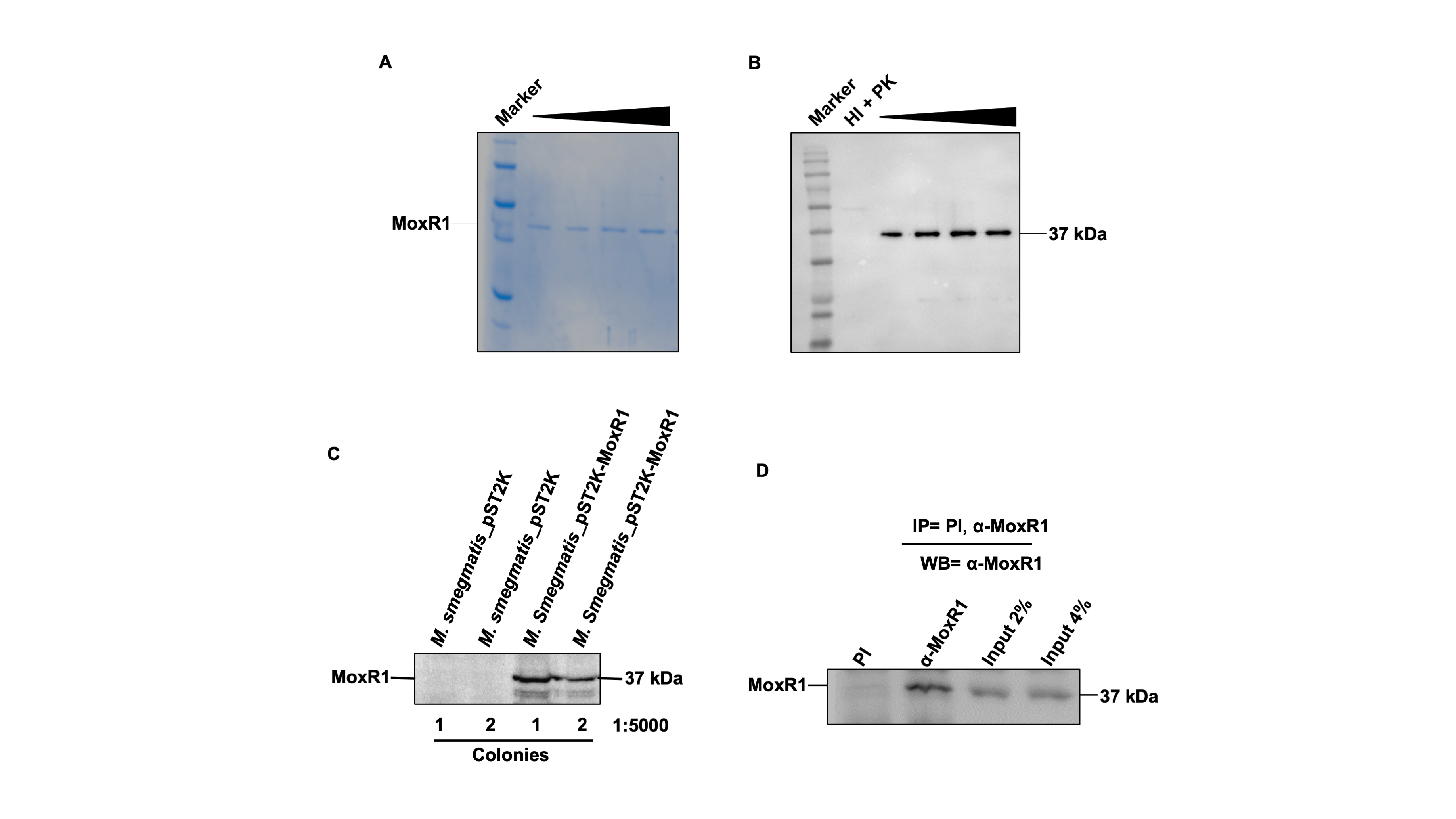

Supplement: Supplemental Material [file KVIR_A_2180230_SM4284.zip › 2180230_-_Supplement/Supp Slide1.tiff]

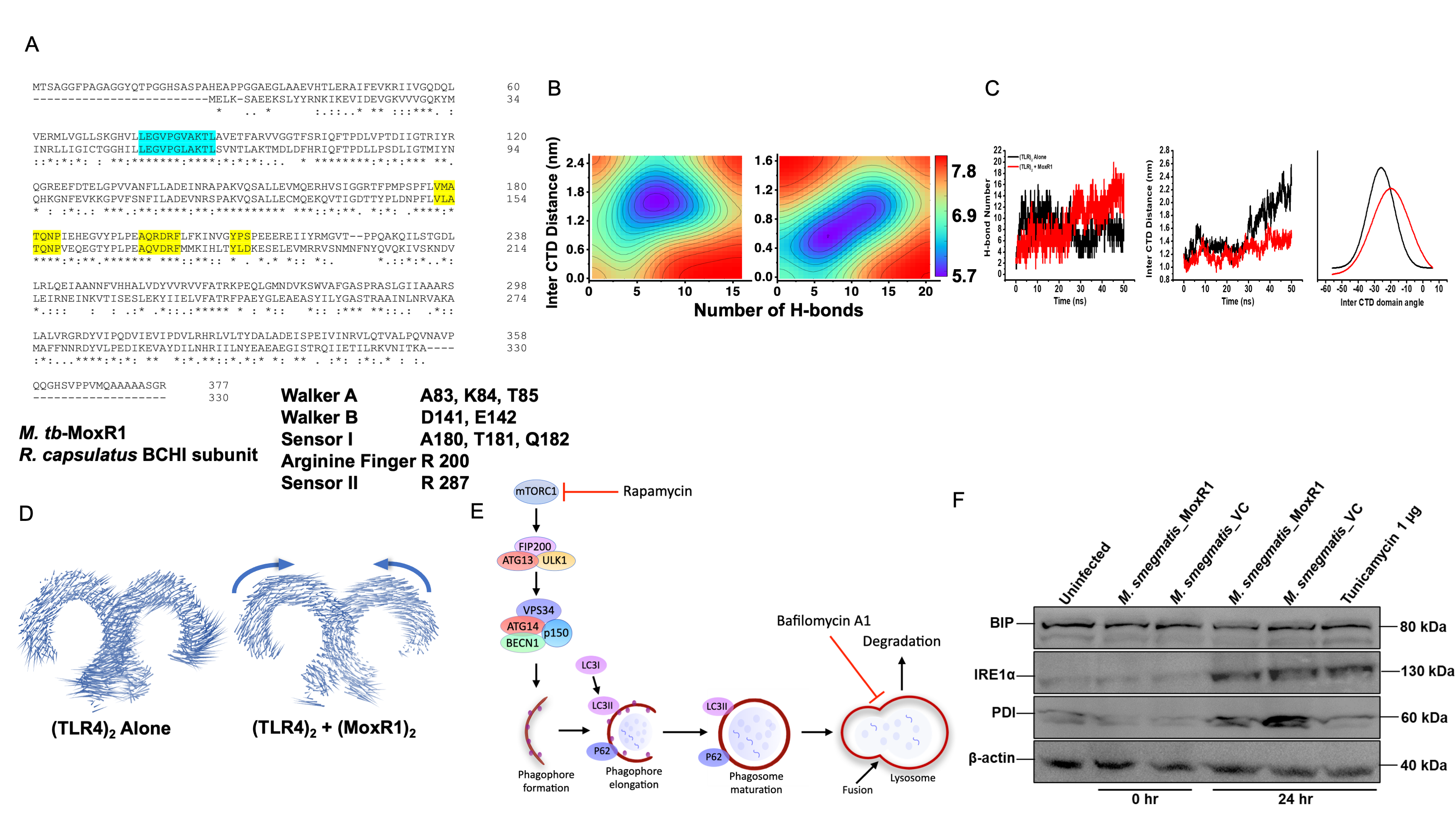

Supplement: Supplemental Material [file KVIR_A_2180230_SM4284.zip › 2180230_-_Supplement/Supp Slide2.tiff]
